# Machine Learning for Predicting the 3-Year Risk of Incident Diabetes in Chinese Adults

**DOI:** 10.3389/fpubh.2021.626331

**Published:** 2021-06-29

**Authors:** Yang Wu, Haofei Hu, Jinlin Cai, Runtian Chen, Xin Zuo, Heng Cheng, Dewen Yan

**Affiliations:** ^1^Department of Endocrinology, The First Affiliated Hospital of Shenzhen University, Shenzhen, China; ^2^Department of Endocrinology, Shenzhen Second People's Hospital, Shenzhen, China; ^3^Shenzhen University Health Science Center, Shenzhen, China; ^4^Department of Nephrology, The First Affiliated Hospital of Shenzhen University, Shenzhen, China; ^5^Department of Nephrology, Shenzhen Second People's Hospital, Shenzhen, China; ^6^Shantou University Medical College, Shantou, China; ^7^Department of Endocrinology, The Third People's Hospital of Shenzhen, Shenzhen, China

**Keywords:** machine learning, extreme gradient boosting, simple stepwise model, Incident diabetes, risk

## Abstract

**Purpose:** We aimed to establish and validate a risk assessment system that combines demographic and clinical variables to predict the 3-year risk of incident diabetes in Chinese adults.

**Methods:** A 3-year cohort study was performed on 15,928 Chinese adults without diabetes at baseline. All participants were randomly divided into a training set (*n* = 7,940) and a validation set (*n* = 7,988). XGBoost method is an effective machine learning technique used to select the most important variables from candidate variables. And we further established a stepwise model based on the predictors chosen by the XGBoost model. The area under the receiver operating characteristic curve (AUC), decision curve and calibration analysis were used to assess discrimination, clinical use and calibration of the model, respectively. The external validation was performed on a cohort of 11,113 Japanese participants.

**Result:** In the training and validation sets, 148 and 145 incident diabetes cases occurred. XGBoost methods selected the 10 most important variables from 15 candidate variables. Fasting plasma glucose (FPG), body mass index (BMI) and age were the top 3 important variables. And we further established a stepwise model and a prediction nomogram. The AUCs of the stepwise model were 0.933 and 0.910 in the training and validation sets, respectively. The Hosmer-Lemeshow test showed a perfect fit between the predicted diabetes risk and the observed diabetes risk (*p* = 0.068 for the training set, *p* = 0.165 for the validation set). Decision curve analysis presented the clinical use of the stepwise model and there was a wide range of alternative threshold probability spectrum. And there were almost no the interactions between these predictors (most *P*-values for interaction >0.05). Furthermore, the AUC for the external validation set was 0.830, and the Hosmer-Lemeshow test for the external validation set showed no statistically significant difference between the predicted diabetes risk and observed diabetes risk (*P* = 0.824).

**Conclusion:** We established and validated a risk assessment system for characterizing the 3-year risk of incident diabetes.

## Highlights

- The eXtreme Gradient Boosting system was an effective machine learning technique.
- We establish a risk assessment system for characterizing the 3-year risk of diabetes.
- The external validation showed that our findings were well-generalized.
- Our findings are helpful for identifying individuals at high risk for diabetes.

## Introduction

The epidemic of diabetes has become a major public health threat across the world. The International Diabetes Federation (IDF) estimated that 451 million adults were suffering from diabetes mellitus worldwide in 2017 and the figure was expected to increase to 693 million by 2045 (1). The prevalence of diabetes among Chinese adults increased from 9.7% in 2007 and to 11.2% in 2017 (2). Diabetes is a debilitating chronic disease with potentially various microvascular and macrovascular complications, such as diabetic kidney disease, diabetic retinopathy, diabetic neuropathy, cardiovascular, and cerebrovascular disease (3–7). Diabetes and its complications have contributed tremendously to the burden of social, financial, and health systems worldwide.

Although diabetes is an irreversible disease, it is largely preventable. Early screening and diagnosis are at the core of effectively preventing diabetes and delaying its progression. Several studies revealed lifestyle modification and pharmacological intervention could reduce the risk of developing diabetes (8, 9). Moreover, for newly diagnosed diabetic patients, intensive lifestyle intervention, early short-term intensive insulin therapy and metabolic surgery can induce long-term glycemic remission without further antidiabetic medication (10–12). Therefore, it is essential to identify individuals at high risk of developing diabetes for diabetes prevention programs.

Machine learning has increasingly been utilized to establish risk prediction models in the field of medicine (13–15). Machine-learning algorithms can be defined as searching through a large number of candidate programs under the guidance of training experience to find a program that optimizes the performance metric (16). Compared with traditional statistical methods, it is mainly applied to iteratively learn the non-linear interactions from a mass of data through computer algorithms (17). Several studies showed that machine learning methods could describe an individual's characteristics and identify individuals at high risk of diabetes (18–21). A gradient tree boosting method implemented in the eXtreme Gradient Boosting (XGBoost) system is an effective machine learning method that can assemble weak prediction models to establish a more reliable prediction model (22–26). So far, there is no research using the XGBoost method to build diabetes risk prediction models. Therefore, we sought to use the XGBoost method to select the most important variables from candidate variables and further establish and validate a risk assessment system that combines demographic and clinical variables using real-world data from a large cohort of Chinese adults across 32 sites and 11 cities between 2010 and 2016 to predict the 3-year risk of incident diabetes in Chinese adults.

## Materials and Methods

### Study Design and Participants

The data was downloaded from the “DATADRYAD” database (www.Datadryad.org), a non-profit computerized database established in China by the Rich Healthcare Group. Its data is available publicly for use. The raw data was provided by Chen et al. (27). The original study recruited a total of 685,277 participants ≥20 years old with at least two visits from 2010 to 2016 across 32 sites and 11 cities in China.

**Figure 1:**
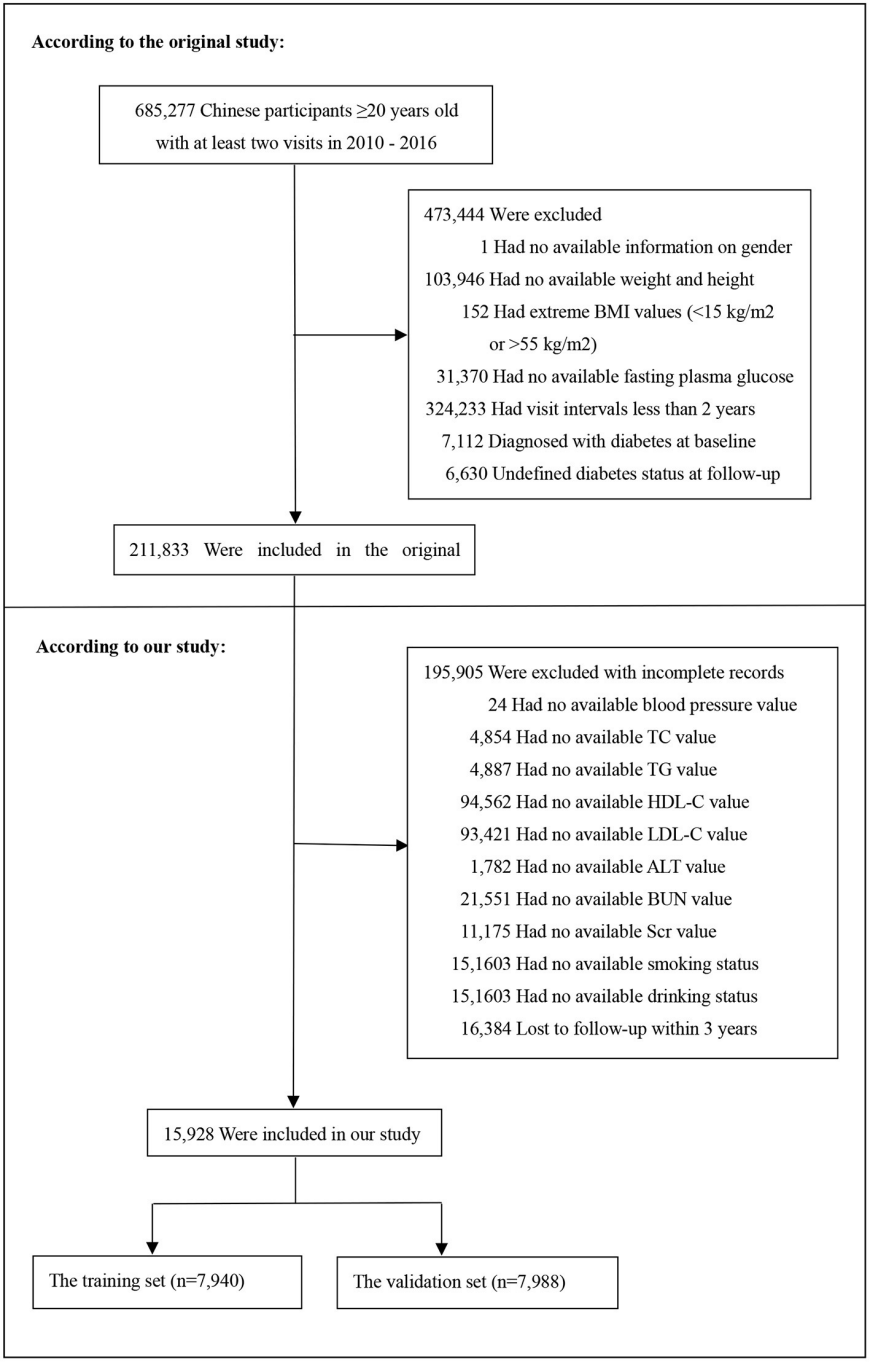
Flowchart of study participants.

Baseline demographic and clinical variables were included as follows: age, gender, smoking and drinking status, family history of diabetes, body mass index (BMI), systolic blood pressure (SBP), diastolic blood pressure (DBP), fasting plasma glucose (FPG), total cholesterol (TC), triglyceride (TG), low density lipoprotein cholesterol (LDL-C), high density lipoprotein cholesterol (HDL-C), serum urea nitrogen (BUN), serum creatinine (Scr), alanine aminotransferase (ALT). The clinical outcome was incident diabetes during a 3-years follow-up. Baseline excluding criteria in the original study included as follows:(1) no available information on weight, height and gender; (2) extreme BMI values (<15 or >55 kg/m^2^); (3) visit intervals <2 years; (4) no available fasting plasma glucose value; (5) participants diagnosed with diabetes at baseline (participants diagnosed by self-report or diagnosed by a fasting plasma glucose ≥7.0 mmol/L) and participants with undefined diabetes status at follow-up. A total of 211,833 participants remained after applying exclusion criteria in the original study. In our study, we further excluded participants with incomplete records. To predicting the 3-year risk of incident diabetes, we also excluded participants who lost to follow-up during 3-years follow up and the censored data is excluded (28). Figure 1 depicted the participants' selection process. Finally, a total of 15,928 subjects (10,313 male and 5,615 female) were included in the present study.

The authors of the original study have waived all copyright and related ownership of the raw data. Therefore, we could use these data for secondary analysis without infringing on the authors' rights. Furthermore, the original study was approved by the Rich Healthcare Group Review Board, and the information was retrieved retrospectively. And the original study was conducted in accordance with the Declaration of Helsinki, so did this secondary research. The data are anonymous, and the requirement for informed consent was waived by the Rich Healthcare Group Review Board due to the observational nature of the study, as reported elsewhere (29).

### Variable Measurement

In each visit to the health check center, participants were required to do a personal questionnaire on demographics, lifestyle, medical history, and family history of chronic disease. And trained staff performed the baseline examination. Weight was measured in light clothing without shoes to the nearest 0.1 kg. The height was accurate to 0.1 cm. BMI was equal to the weight divided by the square of height, which was accurate to 0.1 kg/m^2^. And the staff measured their blood pressure by a standard mercury sphygmomanometer. Fasting venous blood samples were taken after fasting for at least 10 h each visit. Plasma glucose levels were measured by the glucose oxidase method. The clinical measurements of FPG, TC, TG, LDL-C, HDL-C, BUN, Scr, and ALT were conducted by an autoanalyzer (Beckman 5800).

### Definitions

The definitions of diabetes were fasting blood glucose ≥7.00 mmol/L and/or self-reported diabetes during follow-up. Patients were censored either at the time of the diagnosis or at the last visit, whichever comes first.

### Statistical Analysis

All eligible participants were randomly assigned to the training set and the validation set. There were 15 candidate baseline variables involving demographic and clinical characteristics. First, we exclude some variables with relatively significant interference based on collinearity screening. Baseline characteristics were described as means ± standard deviations (normal distribution) or medians (quartiles) (skewed distribution) for continuous variables and as percentages or frequency for categorical variables. We used two-sample *t*-tests to analyze differences between the training set and validation set for normally distributed continuous variables, Wilcoxon rank-sum tests for non-normally distributed continuous variables, and chi-square tests for categorical variables.

XGBoost is a scalable tree boosting system that can assemble weak prediction models to establish a more reliable prediction model (30). During the training process, it can generate a new decision tree through gradient boosting on the basis of the existing decision trees to better predict the results. Therefore, a risk prediction system consisting of a series of decision trees is formed after training. In the application process, the predicted risk output is the cumulative score of each decision tree, representing the probability of the predicted outcome. XGBoost provides the importance score of each variable, indicating the relative number of times the variable is used to distribute data in all trees. We ranked these variables according to the prediction contribution of each variable. Given the Shapley Additive exPlanations (SHAP) approach can transform the original non-linear XGBoost model to the summation effects of all variable attributions while approximating the output risk for each participant (31). Thus, the SHAP method was used to interpret the results of the XGBoost model. We used Shapley values to construct dependency graphs to capture the actual relationship between diabetes risk and the three variables with the most significant prediction contribution. Additionally, we summarized the specificity, sensitivity, accuracy, negative predictive value (NPV), positive predictive value (PPV), positive likelihood ratio (PLR), and negative likelihood ratio (NLR) of the XGBoost model at different predicted probability.

We further established three prediction models based on the predictors chosen by the XGBoost model. First, we applied all risk factors selected by the XGBoost method to build a full model. Second, according to the multivariable fractional polynomials (MFP) algorithm, we used the iterative fashion to determine the significant variables and functional form by backward elimination to establish the MFP model to eliminate the influence of non-linearity and interaction. Third, we conducted a backward step-down selection process based on the Akaike information criterion (AIC) to establish a stepwise model (32). While confirming the statistical significance of the predictor factors, the stepwise logistic regression can achieve local optimal goodness of fit. To assess the discrimination of these risk prediction models, we plotted the receiver operating characteristic (ROC) curve and calculated the area under the ROC curve (AUC) with 95% confidence intervals (CI) for the two sets. Given nomogram is an intuitive graphical prediction model which provides personalized risk predictions for individuals, we further construct the nomogram of the stepwise model. The nomogram is built according to the proportional conversion of each regression coefficient to a 0- to 100-point scale in multiple logistic regression (33). The effect of the variable with the highest β coefficient (absolute value) is assigned 100 points. The point of each variable is added to obtain the total points, which can be converted into the predicted probability of incident diabetes. And we used the Hosmer–Lemeshow test to compare the predicted risk and observed a 3-year incidence of deciles of predicted diabetes risk and we plotted the calibration bar graph of the nomogram for the probability of incident diabetes (34). Besides, we performed decision curve analysis to evaluate the clinical use of the prediction model by quantifying the net benefit at different threshold probabilities: subtracting the proportion of participants with false-positive results from the proportion of participants with true-positive results and then weighing the relative hazards of false positive and false negative results to achieve a net benefit from decision-making (35). And we examined the modifications and interactions between each predictor selected by the stepwise model. In addition, we used a cohort of 11,113 Japanese participants from the NAGALA (NAfd in the Gifu Area, Longitudinal Analysis) database for external validation. The data were also downloaded from the “DATADRYAD” database (www.Datadryad.org), shared by Okamura et al. (36) from: Ectopic fat obesity presents the greatest risk for incident type 2 diabetes: a population-based longitudinal study. Dryad Digital Repository. https://doi.org/10.1038/s41366-018-0076-3. All results are reported in adherence to the TRIPOD statement (37).

All statistical analyses were performed by the statistical software package R (http://www.R-project.org, The R Foundation) and Empower-Stats (http://www.empowerstats.com, X&Y Solutions, Inc., Boston, MA). The tests were 2-tailed, and *P* < 0.05 was taken as statistically significant.

## Results

### Baseline Characteristics of the Study Population

A total of 15,928 eligible participants were included in this study. The mean age of all participants was 43.33 ± 12.31 years old. The male/female ratio was 1.84:1. The mean BMI was 23.53 ± 3.30 Kg/m^2^. The mean FPG was 4.85 ± 0.66 mmol/L. The mean HDL-C and LDL-C were 1.30 ± 0.32 and 2.75 ± 0.69 mmol/L, respectively. TC was excluded based on collinearity screening.

Table 1 compared the baseline characteristics of the training set (*n* = 7,940) and the validation set (*n* = 7,988). After a 3-year follow-up, 148 and 145 incident diabetes cases occurred in the training and validation set, respectively. There were no statistically significant differences in all baseline characteristics and the number of diabetic patients between the two sets (all *P* > 0.05).

**Table 1:** Baseline characteristics of the training and validation sets.

**Characteristic**	**Training set**	**Validation set**	***P*-value**
Participants	7,940	7,988	
Incident diabetes			0.901
No	7,795 (98.17%)	7,840 (98.15%)	
Yes	145 (1.83%)	148 (1.85%)	
Age (year)	43.43 ± 12.45	43.24 ± 12.17	0.339
Gender			0.595
Male	5,157 (64.95%)	5,156 (64.55%)	
Female	2,783 (35.05%)	2,832 (35.45%)	
BMI (kg/m^2^)	23.51 ± 3.28	23.54 ± 3.32	0.552
SBP (mmHg)	119.90 ± 16.00	119.62 ± 15.77	0.266
DBP (mmHg)	75.12 ± 10.46	75.04 ± 10.38	0.633
FPG (mmol/L)	4.86 ± 0.66	4.84 ± 0.66	0.247
TG (mmol/L)	1.17 (0.80–1.77)	1.16 (0.80–1.75)	0.287
HDL-C (mmol/L)	1.30 ± 0.31	1.30 ± 0.33	0.198
LDL-C (mmol/L)	2.75 ± 0.69	2.75 ± 0.69	0.913
ALT (U/L)	20.00 (14.00–30.00)	20.00 (14.00–30.30)	0.566
BUN (mmol/L)	4.66 ± 1.17	4.67 ± 1.16	0.880
Scr (μmol/L)	72.04 ± 15.07	72.11 ± 15.25	0.767
Smoking status			0.443
Ever/current	1972 (24.84%)	2026 (25.36%)	
Never	5968 (75.16%)	5962 (74.64%)	
Drinking status			0.624
Ever/current	1,544 (19.45%)	1,578 (19.75%)	
Never	6,396 (80.55%)	6,410 (80.25%)	
Family history			0.157
No	7,400 (93.20%)	7489 (93.75%)	
Yes	540 (6.80%)	499 (6.25%)	

*Values are n (%) or mean ± SD*.

*BMI, Body mass index; SBP, Systolic blood pressure; DBP, Diastolic blood pressure; FPG; Fasting plasma glucose; TG, Triglyceride; HDL-C, High density lipoprotein cholesterol; LDL-C, Low density lipid cholesterol; ALT, Alanine aminotransferase; BUN, Blood urea nitrogen; Scr, Serum creatinine; Family history, Family history of diabetes*.

### Development of XGBoost Model

**Figure 2:**
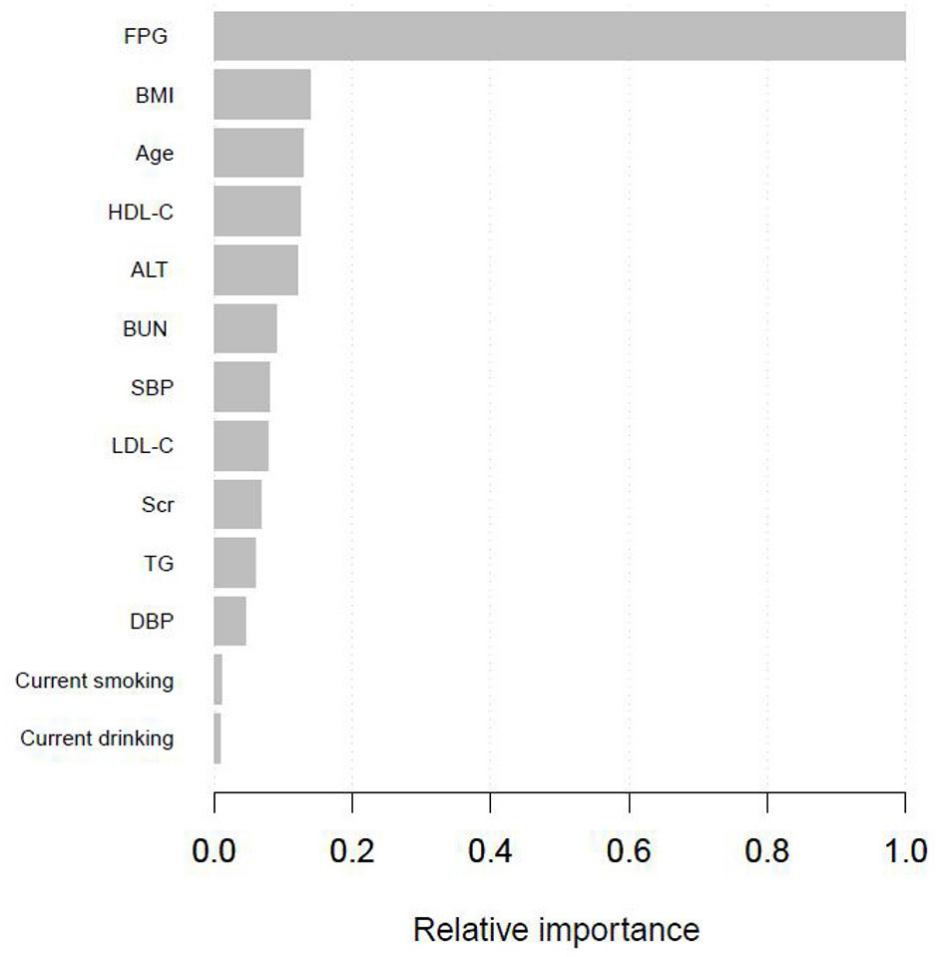
Shapley values-based interpretation of the model. Contributing feature importance of the variables selected by the XGBoost model.

Supplementary Table 1 presented the variables selected by the XGBoost model and the corresponding prediction contributions. The XGBoost model incorporated FPG, BMI, age, HDL-C, ALT, BUN, SBP, LDL-C, Scr, TG, DBP, current smoking, and drinking. The importance score of FPG was 0.5125 and its relative importance was 1.0000, which was the most important variable. The importance score of BMI was 0.0708 and its relative importance was 0.1382, and its prediction contribution was only lower than that of FPG. And the importance score of age is 0.0658, ranking third in the prediction contributions. Figure 2 showed the ranking of the variables based on contributing features. Supplementary Figure 1 demonstrated the discriminatory ability of the XGBoost model. The AUCs of the XGBoost model in the training set and validation set were 0.977 and 0.920, respectively. Given FPG, BMI and age shared the top 3 contributing features, and we further used the SHAP method to explore the actual relationship between diabetes risk and them (Supplementary Figure 2). When FPG <4.6 mmol/L, the risk of incident diabetes was at a low level. However, when FPG > 4.6 mmol/L, with the increase of FPG, the risk of developing diabetes increased rapidly. And as BMI and age increased, the risk of diabetes gradually increased.

Supplementary Figure 3 presented the result of the decision curve analysis for the XGBoost model. The results showed that if the personal threshold probability of a participant is 50% (i.e., the participant would opt for diabetes screening if the probability of incident diabetes was <50%), then the net benefit is 0.453 when using the model to decide whether to perform diabetes screening (i.e., oral glucose tolerance test), with added benefit compared to the diabetes screening for all or none participants.

And Supplementary Table 2 summarized the sensitivity and specificity for predicting incident diabetes at different cutoff values in the XGBoost model. The result showed that although higher cutoff values lead to higher specificity, the sensitivity rapidly dropped to a relatively low level.

### Construction of the Stepwise Model

**Figure 3:**
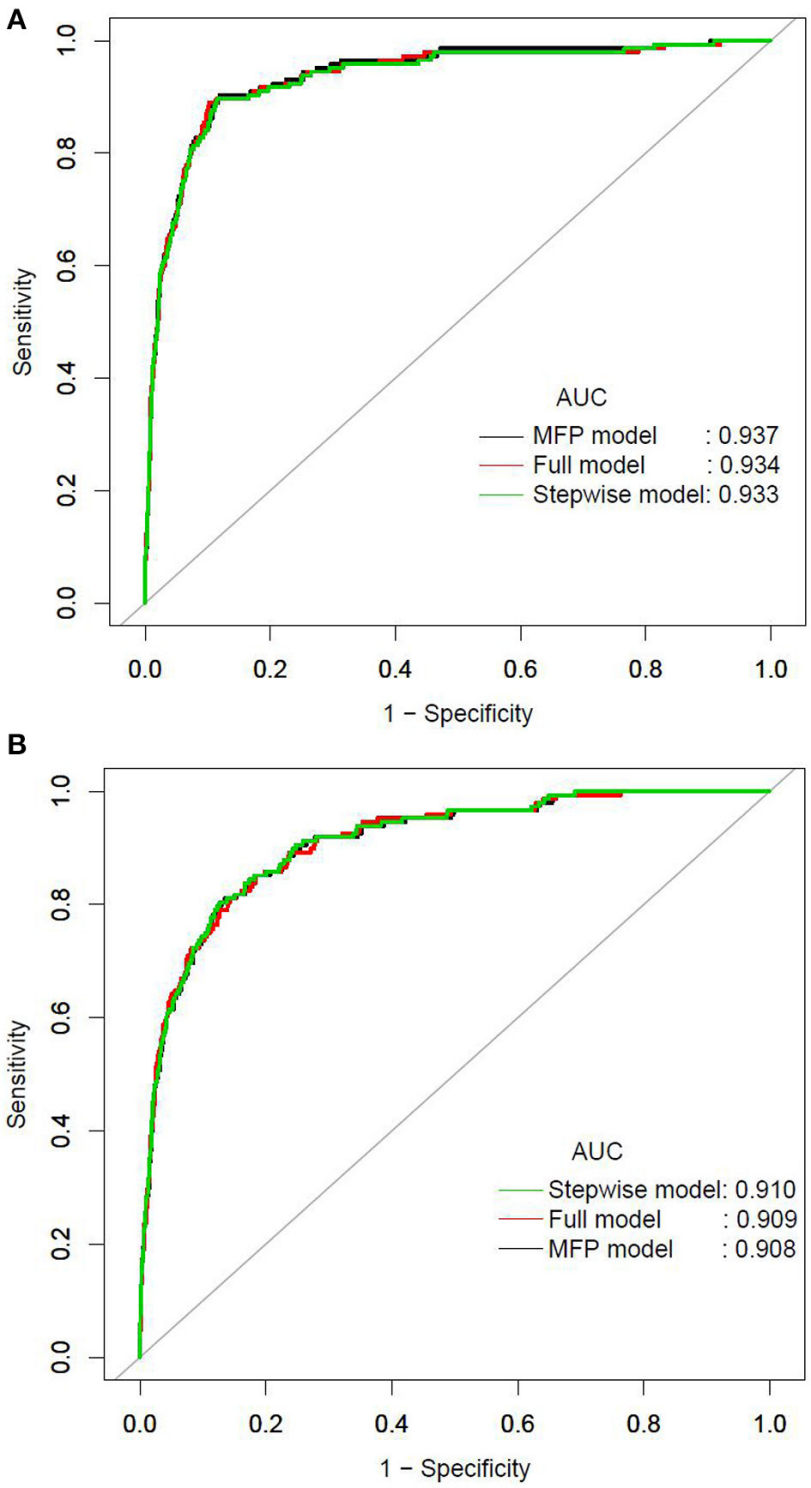
The ROC curves of the MFP model, full model and stepwise model in the training set **(A)** and validation set **(B)**.

**Figure 4:**
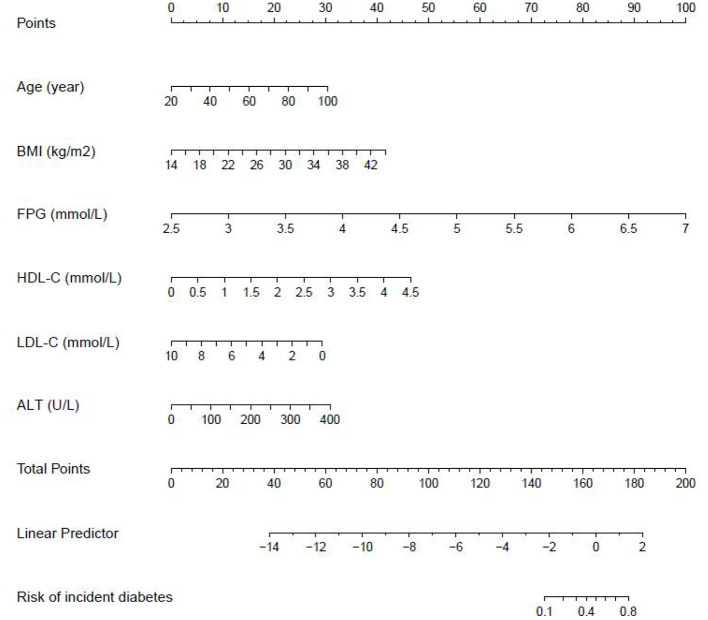
The nomogram of the stepwise model to predict the 3-year risk of incident diabetes. When predicting an individual's 3-year risk of diabetes, locate his/her value on each variable axis. Draw a vertical line from that value to the top Points scale to determine how many points are assigned by that variable value. Then, the points from each variable value are summed. Locate the sum on the Total Points scale and vertically project it onto the bottom axis, thus obtaining a personalized 3-year risk of diabetes.

We further established three prediction models based on the predictors chosen by the XGBoost model, including the MFP model, full model and stepwise model. In the training set, AUCs of the MFP model, full model and stepwise model were 0.937, 0.934 and 0.933, respectively. In the validation set, the corresponding AUCs of those models were 0.908, 0.909 and 0.910, respectively (Figure 3, Supplementary Table 3). The AUCs of the three models were relatively close. Given that the stepwise model incorporated fewer risk factors and it was simpler than MFP and full models. Besides, the stepwise model could predict the 3-year diabetes risk relatively well. Therefore, we chose the stepwise model as the optimal risk prediction model for incident diabetes. Table 2 showed the 6 variables were selected by stepwise model, including FPG, BMI, age, HDL-C, ALT, and LDL-C. The results showed FPG, BMI, age, HDL-C and ALT were positively associated with incident diabetes. And participants with relatively high FPG were more likely to develop diabetes [relative risk (RR):11.2812; 95% CI: 8.0798–16.4983]. In contrast, participants with relatively high LDL-C were less likely to develop diabetes (RR, 0.7238; 95% CI: 0.5438–0.9229). We further draw a corresponding nomogram to provide a quantitative and simple tool in predicting the risk of diabetes by using age, BMI, FPG, HDL-C, LDL-C, and ALT (Figure 4). Each variable in the nomogram was assigned a specific point, and the points from each variable value are summed to obtain the total points, which was used to obtain the probability for predicting diabetes. And the algorithm of diabetes risk in stepwise model was logit (risk of incident diabetes) = −24.07232 +0.04191^*^age (year) + 0.15291^*^BMI (kg/m^2^) + 2.45073^*^FPG (mmol/L) + 1.14025^*^HDL-C (mmol/L) - 0.32400^*^LDL-C (mmol/L) + 0.00852^*^ALT (U/L).

**Table 2:** Variables selected using stepwise logistic regression.

	**Beta**	**Standard error**	***z*-value**	**RR (95%CI)**	***P*-value**
(Intercept)	−24.07232	1.34753	−17.86405	–	–
FPG (mmol/L)	2.45073	0.15763	15.54774	11.2812 (8.0798–16.4983)	0.0000
HDL-C (mmol/L)	1.14025	0.29593	3.85313	3.1101 (1.7651–5.8612)	0.0000
BMI (kg/m^2^)	0.15291	0.03016	5.07010	1.1647 (1.0911–1.2413)	0.0000
Age (year)	0.04191	0.00765	5.47752	1.0427 (1.0276–1.0578)	0.0000
ALT (U/L)	0.00852	0.00335	2.53939	1.0085 (1.0022–1.0146)	0.0060
LDL-C (mmol/L)	−0.32400	0.14526	−2.23050	0.7238 (0.5438–0.9229)	0.0030

*FPG; Fasting plasma glucose; HDL-C, High density lipoprotein cholesterol; BMI, Body mass index; LDL-C, Low density lipid cholesterol; ALT, Alanine aminotransferase; RR, Relative risk; CI, Confidence interval*.

### Performance of the Stepwise Model

**Figure 5:**
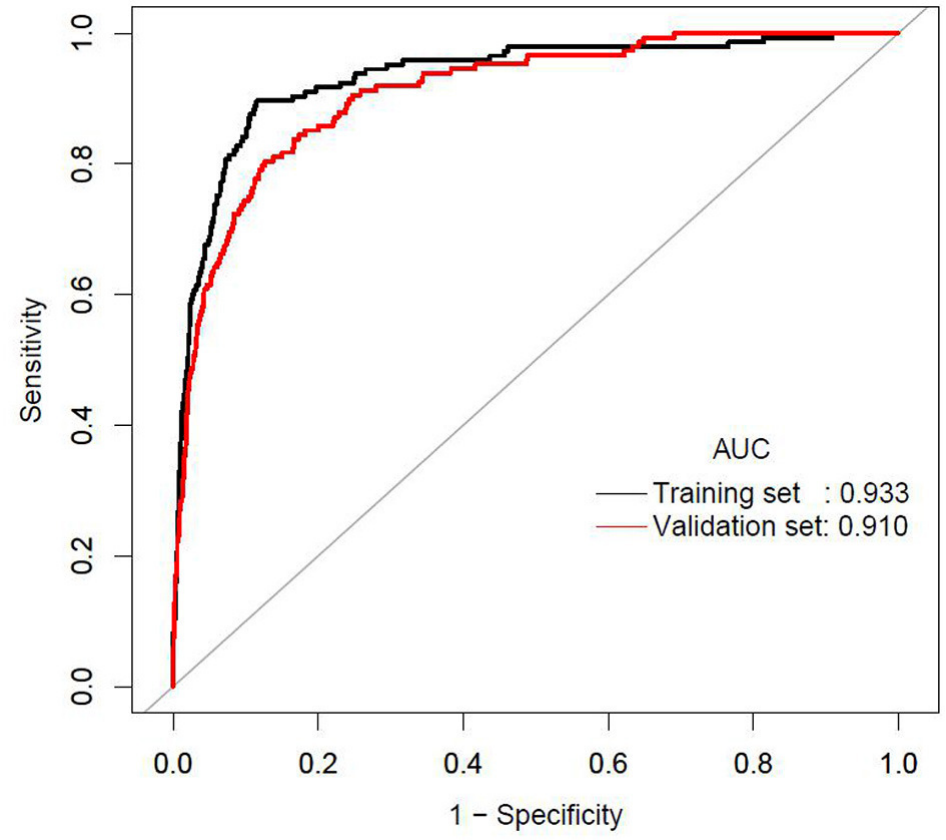
The ROC curves of the stepwise model in the training set and validation set.

**Figure 6:**
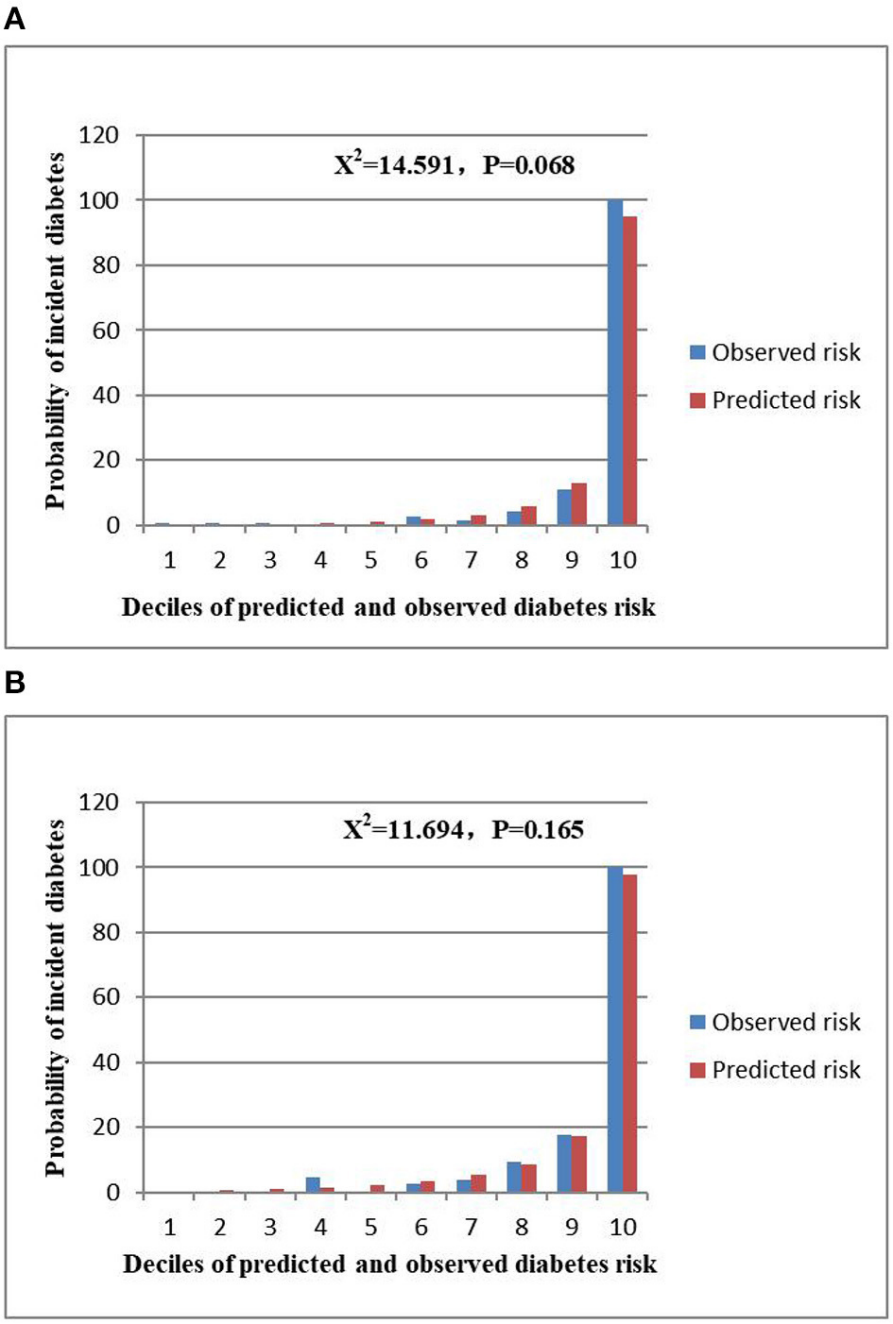
Comparison between predicted and observed 3-year incidence of deciles of the predicted diabetes risk score in the nomogram for the training set **(A)** and validation set **(B)**.

The AUCs of the stepwise model were 0.933 and 0.910 in the training and validation sets, respectively (Figure 5). And the result of bootstrap resampling validation (times = 500) confirmed that the prediction performance of the stepwise model in the training cohort was stable (AUC = 0.927) (Supplementary Figure 4). The calibration bar graph of the nomogram for the probability of incident diabetes demonstrated good agreement between observation and prediction both in the training and validation sets (Figure 6). The Hosmer-Lemeshow test indicated that the model was non-significant (*p* = 0.068 for the training set, *p* = 0.165 for the validation set), suggesting a perfect fit between the predicted diabetes risk and the observed diabetes risk.

**Figure 7:**
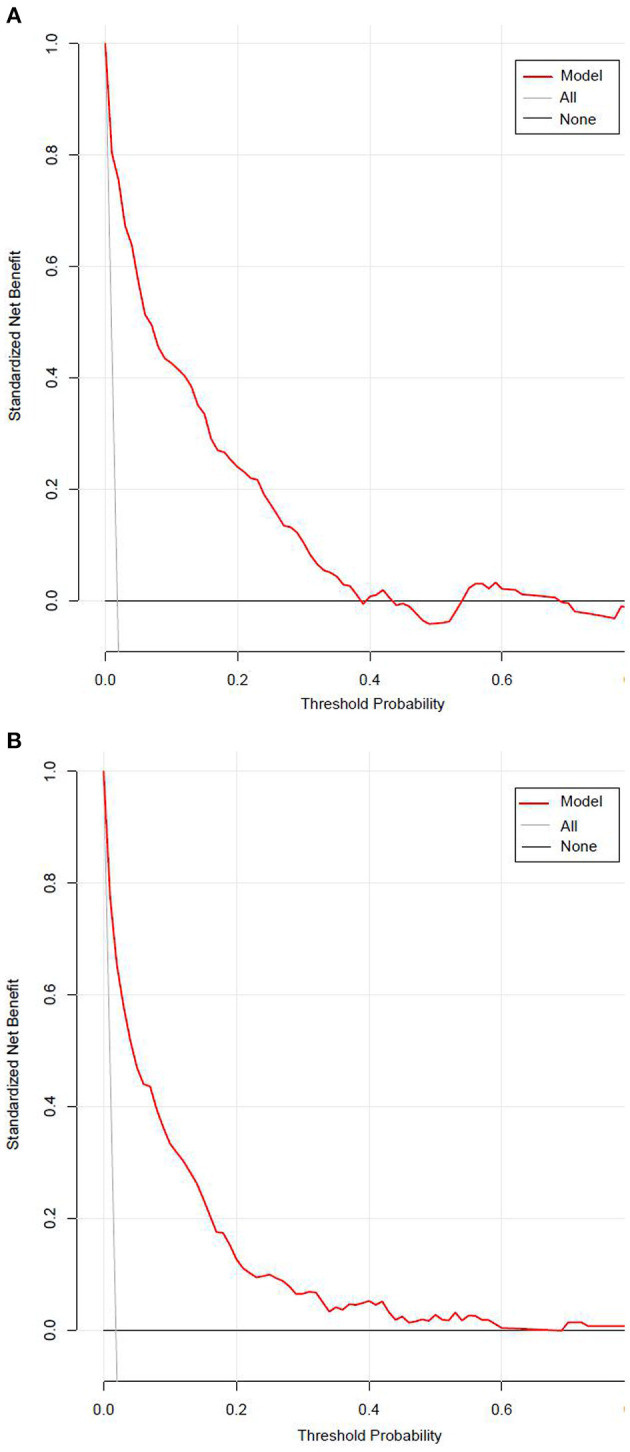
The decision curve for the stepwise model predicts the risk of incident diabetes in the training set **(A)** and validation set **(B)**. Net benefit is shown on the y-axis. The red line represents the model; the thin gray line represents the assumption that all participants develop diabetes; the thin black line represents the assumption that none participants develop diabetes. The decision curve demonstrated that if the threshold probability of a patient is >1%, using the model to predict incident diabetes adds more benefit than diabetes screenings (i.e., oral glucose tolerance test) for all or none of the participants.

Figure 7 presented the result of decision curve analysis for the stepwise model. The decision curve demonstrated if the threshold probability of a patient was >1%, using the XGBoost model to predict incident diabetes was more beneficial than diabetes screening for all or none of the participants. There was a wide range of alternative threshold probability spectrum, which indicated that the stepwise model had significant clinical use.

### Modifications and Interactions Between Each Predictor in the Nomogram

We examined the modifications and interactions between each predictor selected by the stepwise model, including age, BMI, FPG, HDL-C, LDL-C, and ALT. Table 3 showed that almost no interactions were observed based on our prior specification (most *P*-values for interaction >0.05), except that BMI and FPG had significant interactions (*P*-values for interaction = 0.017).

**Table 3:** Modifications and interactions between each predictor selected by the stepwise model.

**Predictor**	**Modifier**	**HR (95%CI)**	***P* for interaction**
Age	BMI	0.997 (0.994, 1.001)	0.186
Age	FPG	0.980 (0.958, 1.002)	0.077
Age	ALT	1.000 (0.999, 1.000)	0.824
Age	HDL-C	1.015 (0.969, 1.064)	0.524
Age	LDL-C	0.996 (0.974, 1.018)	0.699
ALT	FPG	1.001 (0.991, 1.011)	0.902
ALT	BMI	1.000 (0.999, 1.002)	0.627
ALT	HDL-C	0.999 (0.979, 1.019)	0.896
ALT	LDL-C	0.994 (0.986, 1.002)	0.148
BMI	FPG	0.904 (0.832, 0.982)	0.017
BMI	HDL-C	0.978 (0.840, 1.139)	0.776
BMI	LDL-C	1.001 (0.923, 1.086)	0.979
FPG	HDL-C	1.903 (0.692, 5.233)	0.213
FPG	LDL-C	1.034 (0.643, 1.665)	0.889
HDL-C	LDL-C	1.268 (0.560, 2.872)	0.569

*FPG; Fasting plasma glucose; HDL-C, High density lipoprotein cholesterol; BMI, Body mass index; LDL-C, Low density lipid cholesterol; ALT, Alanine aminotransferase; HR, Hazard Ratio; CI, Confidence interval*.

### External Validation

**Figure 8:**
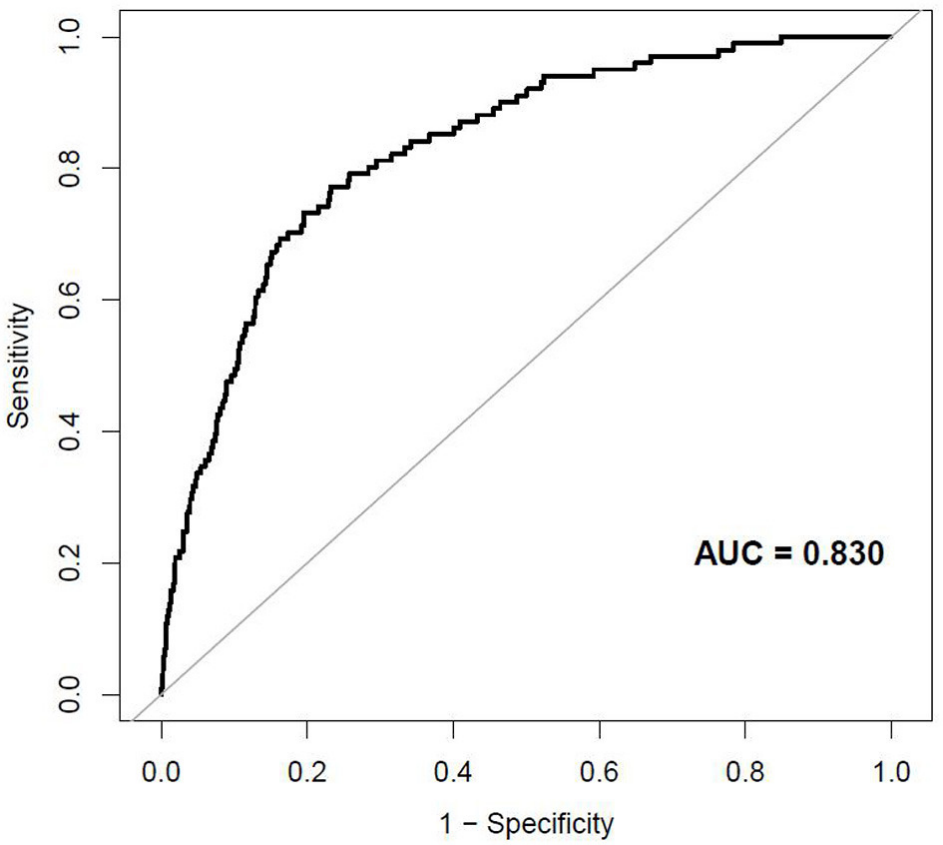
The ROC curves of the external validation.

**Figure 9:**
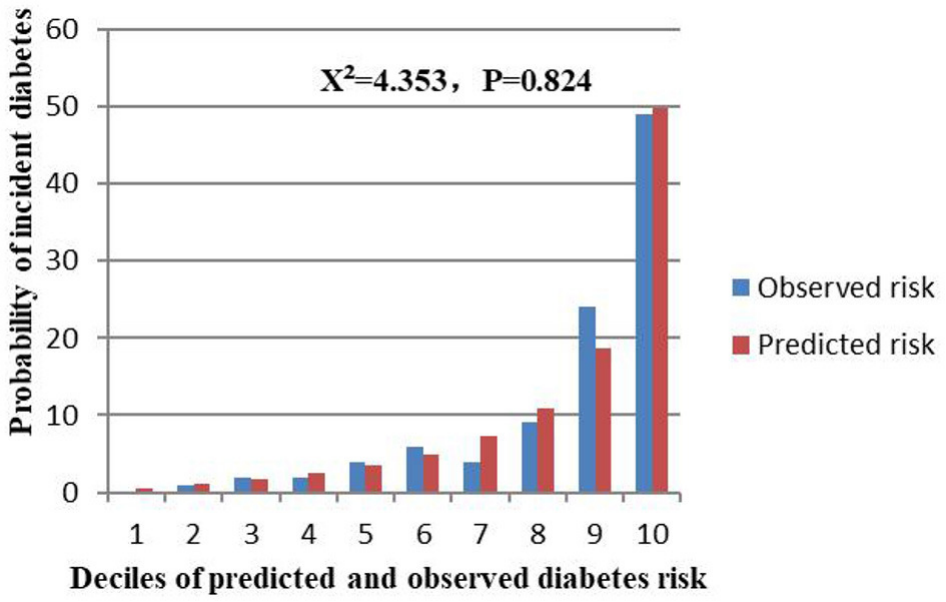
Comparison between predicted and observed 3-year incidence of deciles of a predicted diabetes risk score for the external validation set.

The external validation was performed on a cohort of 11,113 Japanese participants. The AUC for the external validation set was 0.830, which showed good discrimination (Figure 8). And the Hosmer-Lemeshow test for the external validation set showed no statistically significant difference between the predicted diabetes risk and observed diabetes risk, which revealed a perfect fit between the predicted diabetes risk and the observed diabetes risk (*P* = 0.824) (Figure 9). In short, the external validation indicated that the stepwise model was well-generalized.

## Discussion

In the present study, we established and validated a risk assessment system for characterizing the 3-year risk of incident diabetes. The XGBoost model incorporated FPG, BMI, Age, HDL-C, ALT, BUN, SBP, LDL-C, Scr, TG, DBP, current smoking, and drinking, of which FPG, BMI and age shared the top three prediction contribution. And we further established a stepwise model and a corresponding prediction nomogram based on the predictors chosen by the XGBoost model. The AUCs of the stepwise model were 0.933 and 0.910 in the training and validation sets, respectively. The Hosmer-Lemeshow test showed a perfect fit between the predicted diabetes risk and the observed diabetes risk (*p* = 0.068 for the training set, *p* = 0.165 for the validation set). Decision curve analysis presented the clinical use of the stepwise model and there was a wide range of alternative threshold probability spectrum. Furthermore, the AUC for the external validation set was 0.830, and the Hosmer-Lemeshow test for the external validation set showed no statistically significant difference between the predicted diabetes risk and observed diabetes risk (*P* = 0.824). Therefore, the external validation indicated that the stepwise model was well-generalized.

Machine learning is a collection of data analysis techniques, which aims to establish prediction models that improve with experience and it is becoming an important part of modern medical research (13). It holds promise to enable computers to assist humans to analyze large and complex data sets (14). So far, researchers have developed a variety of machine learning algorithms, including decision trees, kernel machines, neural networks, support vector machines, logistic regression, Bayesian classifiers, ensemble learning, multilayer perceptron, and so on (38–45). Machine learning has unique advantages, including scalability and flexibility, making it applicable to various tasks, such as classification, risk stratification, diagnosis and survival predictions (46). Besides, it handles large multidimensional sets of time-to-event data without the need for assumptions of normality of distributions, linearity of risk prediction, and overfitting of models (47). As yet, machine learning techniques have been applied to a broad range of areas within diabetes, some of which are used to build risk prediction models for incident diabetes (20, 21, 48–52). As a novel machine learning method, XGBoost has become well-established in the machine learning community and gained a positive reputation through numerous machine learning challenges (53). The XGBoost algorithm can automatically handle missing data by adding a default direction for the missing values in each tree node (54). XGBoost has higher calculating speed and accuracy based on the principle of gradient boosting (30). Evidence showed that XGBoost's performance was significantly better than traditional statistical methods (24, 55, 56). To our knowledge, the XGBoost method has not been applied to develop a diabetes risk prediction model. In addition, in those studies using machine learning techniques to predict the risk of diabetes, researchers mainly focused on comparing various machine learning methods but did not extend the results of machine learning to clinical applications. And AUCs of those models were between 0.580 and 0.925 (20, 21, 48–52). However, this was the first study that used XGBoost method to evaluate the importance of variables and characterize the 3-year risk of incident diabetes among Chinese adults. Notably, we ranked the variables according to the prediction contribution of each selected variable. Furthermore, we used the SHAP method to capture the actual relationship between diabetes risk and the three variables with the largest predicted contribution. Moreover, we developed a simple stepwise model and constructing a corresponding nomogram based on the XGBoost model. And we performed the Hosmer-Lemeshow test to explore the difference between the predicted diabetes risk and the observed diabetes risk. And we did the decision curve analysis to explore the clinical use of the stepwise model, and there was a wide range of alternative threshold probability spectrum. Moreover, we examined the modifications and interactions between each predictor selected by the stepwise model. Furthermore, we used a cohort of 11,113 Japanese participants as the external validation set to explore the reliability and generalizability of the stepwise model.

Diabetes can cause various complications, bring severe physical and psychological distress to patients, and bring a huge burden to the healthcare system. And it tends to be undiagnosed due to the lack of specific symptoms. However, screening for diabetes through oral glucose tolerance test may increase the yield and economic efficiency of screening (57). Our results made up for this deficiency, which helps identify individuals with a high risk of developing diabetes and avoiding the costs and efforts of prevention and treatment in low-risk groups.

Identifying key factors has great clinical significance in the risk assessment of incident diabetes. FPG was the most important risk predictor in our study. Impaired fasting plasma glucose is one of the diagnostic criteria for diabetes. Researchers found compared with those with impaired fasting blood glucose, people with normal fasting blood glucose have a significantly lower risk of developing diabetes (4.0 vs. 11.3%) (58). BMI had the second-largest predicted contribution. The original research showed for every 1 kg/m^2^ increase in BMI among Chinese adults, the risk of diabetes increases by 23% (27). Multiple studies have demonstrated overweight or obesity was related to the risk of diabetes (59, 60). Evidence showed obesity, dyslipidemia, abnormal hepatocellular function, and diabetes usually coexist in the same subject and have common pathological mediators (inflammation, metabolic disorders, insulin resistance and intestinal flora imbalance, etc.) (61–63). The prevalence of diabetes markedly increases with age (64). The aging of pancreatic β cells can lead to decreased glucose sensitivity and insulin secretion defects (65). Therefore, the application of these risk predictors in our models is well-founded.

There are some strengths of our study, as follows: (1) As a large-scale multicenter study, our models can be well-applied to the Chinese population. (2) This was the first study that used the XGBoost method to characterize the 3-year risk of incident diabetes. (3) We presented the predicted contribution of each variable selected by the XGBoost model and sorted them in the form of a bar chart. (4) We developed a simple stepwise model based on the XGBoost model and constructed a corresponding nomogram to provide a personalized risk assessment tool. (5) We examined the modifications and interactions between each predictor selected by the stepwise model. (6) We used a cohort of Japanese participants as the external validation set to explore the reliability and generalizability of the stepwise model. (7) Since this was a retrospective cohort study, it could decrease the risk of selection bias and observation bias.

However, there are still some potential limitations. First, the variables we extracted were limited and lacked information about other diabetes risk factors, such as glycated glycosylated hemoglobin, serum insulin and C-peptide concentration. Second, due to the original study design, we cannot distinguish the types of diabetes mellitus. Considering type 2 diabetes mellitus is the most common kind of diabetes, accounting for over 90% of diabetes cases (66), our findings represent type 2 diabetes mellitus. Third, the researchers did not perform a 2-h oral glucose tolerance test. Thus, our diagnostic criteria for diabetes mellitus may have missed some diabetic patients. However, it is not feasible to perform an oral glucose tolerance test on all participants in such a large-scale cohort study. Fourth, there are too many missing values of variables in the original data, and multiple imputations to replace missing values were not feasible. Therefore, we excluded participants with incomplete records for a complete case study.

## Conclusion

We established and validated a risk assessment system for characterizing the 3-year risk of incident diabetes, which showed outstanding performance. And FPG, BMI and age shared the top three prediction contributions. We also constructed a prediction nomogram to provide a personalized risk assessment tool for developing diabetes.

## Data Availability Statement

The original contributions presented in the study are included in the article/Supplementary Materials, further inquiries can be directed to the corresponding author.

## Ethics Statement

The studies involving human participants were reviewed and approved by the Rich Healthcare Group Review Board,and the information was retrieved retrospectively.The data are anonymous, and the requirement for informed consent was waived by the Rich Healthcare Group Review Board due to the observational nature of the study, as reported elsewhere.

## Author Contributions

YW and HH conceived and designed the research and drafted the manuscript. JC and RC did statistical analysis. XZ and HC took part in the discussion. DY revised the manuscript. All authors read and approved the final manuscript.

## Conflict of Interest

The authors declare that the research was conducted in the absence of any commercial or financial relationships that could be construed as a potential conflict of interest.

## Abbreviations

BMI: Body mass index
SBP: Systolic blood pressure
DBP: Diastolic blood pressure
FPG: Fasting plasma glucose
TC: Total cholesterol
TG: Triglyceride
HDL-C: High-density lipoprotein cholesterol
LDL-C: Low-density lipid cholesterol
ALT: Alanine aminotransferase
BUN: Serum urea nitrogen
Scr: Serum creatinine
Family history: Family history of diabetes
XGBoost: EXtreme Gradient Boosting
SHAP: Shapley Additive exPlanations
SD: Standardized difference
RR: Relative risk
CI: Confidence intervals
PPV: Positive predictive value
NPV: Negative predictive value
PLR: Positive likelihood ratio
NLR: Negative likelihood ratio
DOR: Diagnostic odds ratio
ROC: Receiver operating characteristic
AUC: Area under curve.
