# Supplementary material for: Machine Learning for Predicting the 3-Year Risk of Incident Diabetes in Chinese Adults
Source: Front Public Health. 2021 Jun 29;9:626331. doi: 10.3389/fpubh.2021.626331 (PMC8275929; doi:10.3389/fpubh.2021.626331)
Supplement: Supplementary file 1 [file Data_Sheet_1.pdf]

Table S1 Variables selected by XGBoost and the corresponding variable importance score.

Variable	importance score	Relative importance
**FPG (mmol/L)**	0.5125	1.0000
**BMI (kg/m2)**	0.0708	0.1382
**Age (year)**	0.0658	0.1284
**HDL-C(mmol/L)**	0.0642	0.1253
**ALT(U/L)**	0.0614	0.1199
**BUN (mmol/L)**	0.0466	0.0910
**SBP (mmHg)**	0.0409	0.0798
**LDL-C(mmol/L)**	0.0398	0.0777
**Scr (umol/L)**	0.0344	0.0672
**TG (mmol/L)**	0.0301	0.0588
**DBP (mmHg)**	0.0232	0.0452
**Current smoking**	0.0054	0.0106
**Current drinking**	0.0047	0.0092

BMI, Body mass index; SBP, Systolic blood pressure; DBP, Diastolic blood pressure; FPG; Fasting plasma glucose; TG, Triglyceride; HDL-C, High density lipoprotein cholesterol; LDL-C, Low density lipid cholesterol; ALT, Alanine aminotransferase; BUN, Blood urea nitrogen; Scr, Serum creatinine;

Table S2 Prediction performance of the XGBoost prediction model at different cutoff values.

Predicted Probability	Specificity (%)	Sensitivity (%)	Accuracy (%)	PPV (%)	NPV (%)	PLR	NLR
**≥0.05**	98.56	77.24	98.17	50.OO	99.57	53.76	0.23
**≥0.1**	99.03	73.79	98.56	58.47	99.51	75.69	0.26
**≥0.2**	99.38	66.21	98.78	66.67	99.37	107.52	0.34
**≥0.3**	99.55	63.45	98.89	72.44	99.32	141.31	0.37
**≥0.4**	99.70	57.93	98.94	78.50	99.22	196.34	0.42
**≥0.5**	99.76	57.24	98.98	81.37	99.21	234.84	0.43
**≥0.6**	99.81	56.55	99.02	84.54	99.20	293.88	0.44
**≥0.7**	99.85	53.79	99.01	86.67	99.15	349.43	0.46
**≥0.8**	99.92	51.03	99.03	92.50	99.10	663.02	0.49
**≥0.9**	99.99	40.69	98.90	98.33	98.91	3171.76	0.59

PPV, Positive predictive value; NPV, Negative predictive value; PLR, Positive likelihood ratio; NLR, Negative likelihood ratio.

Table S3 Prediction performance of MFP, full and stepwise model for the risk of diabetes.

	Training set			Validation set		
	MFP model	Full model	Stepwise model	MFP model	Full model	Stepwise model
AUC	0.9373	0.9341	0.9328	0.9082	0.9090	0.9097
95% CI						
Lower	0.9163	0.9114	0.9100	0.8844	0.8852	0.8863
Upper	0.9582	0.9569	0.9557	0.9320	0.9327	0.9332
Best threshold	0.0214	0.0259	0.0226	0.0181	0.0118	0.0197
Specificity, %	88.02	89.80	88.48	86.51	81.57	87.31
Sensitivity, %	90.34	88.97	89.66	81.08	85.14	80.41
Accuracy, %	88.06	89.79	88.50	86.40	81.63	87.18
PPV, %	12.30	13.96	12.65	10.19	8.02	10.68
NPV, %	99.80	99.77	99.78	99.59	99.66	99.58
PLR	7.5400	8.7231	7.7824	6.0083	4.6191	6.3355
NLR	0.1097	0.1229	0.1169	0.2187	0.1822	0.2244
DOR	68.7359	70.9906	66.5635	27.4723	25.3466	28.2292

MFP, multivariable fractional polynomials; AUC, Area under curve; CI, Confidence interval; PPV, Positive predictive value; NPV, Negative predictive value; PLR, Positive likelihood ratio; NLR, Negative likelihood ratio; DOR, Diagnostic odds ratio.

Fig S1

A

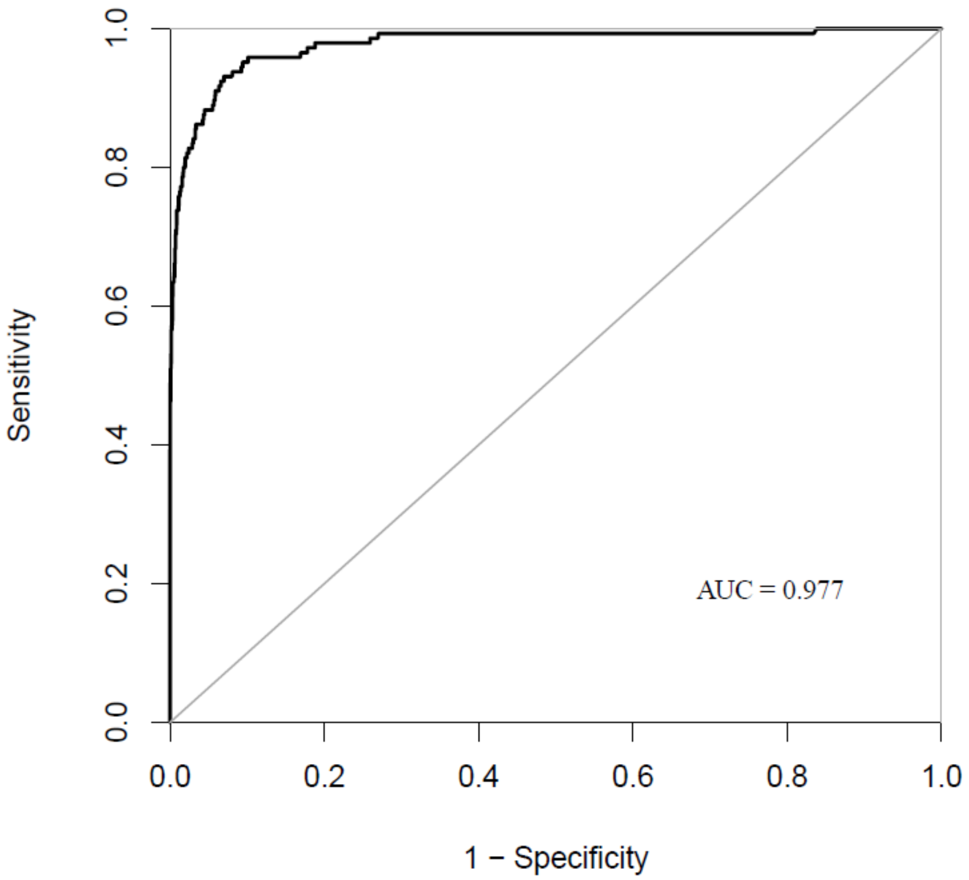
1.0
0.8
0.6
0.4
0.2
0.0
Sensitivity
AUC = 0.977
0.0
0.2
0.4
0.6
0.8
1.0
1 − Specificity

Fig S1

B

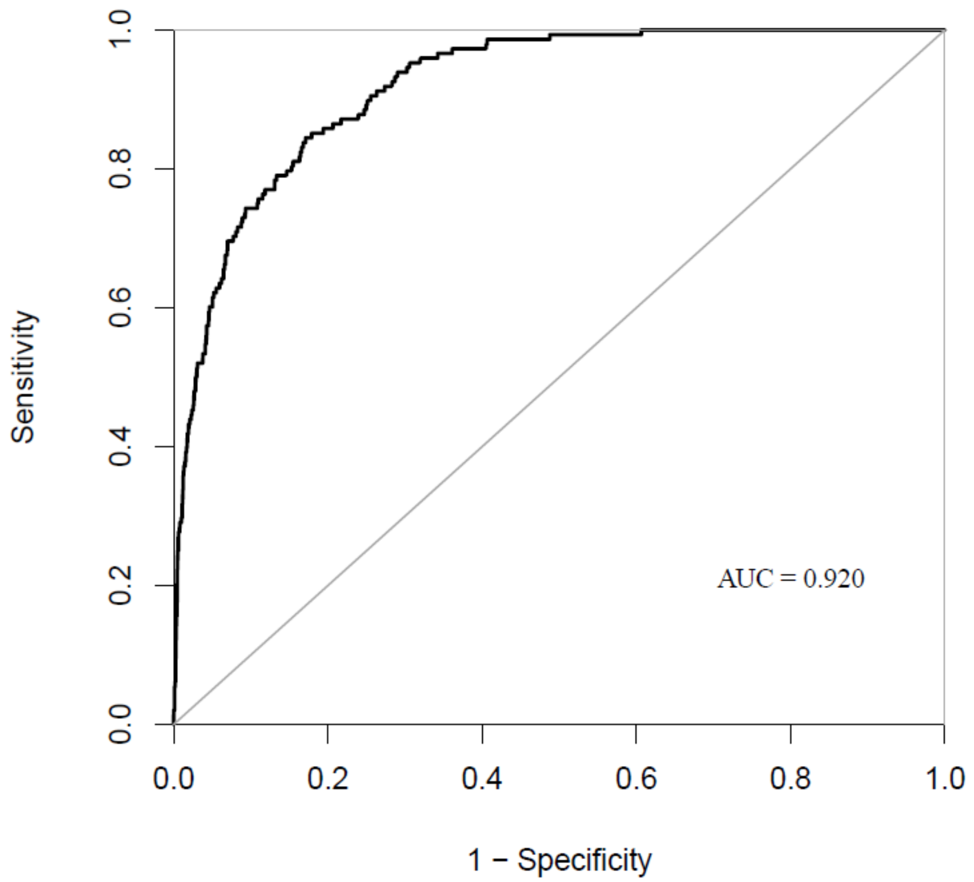

Fig S1. The ROC curves of the XGBoost model in the training set (a) and validation set (b).

Fig S2

A

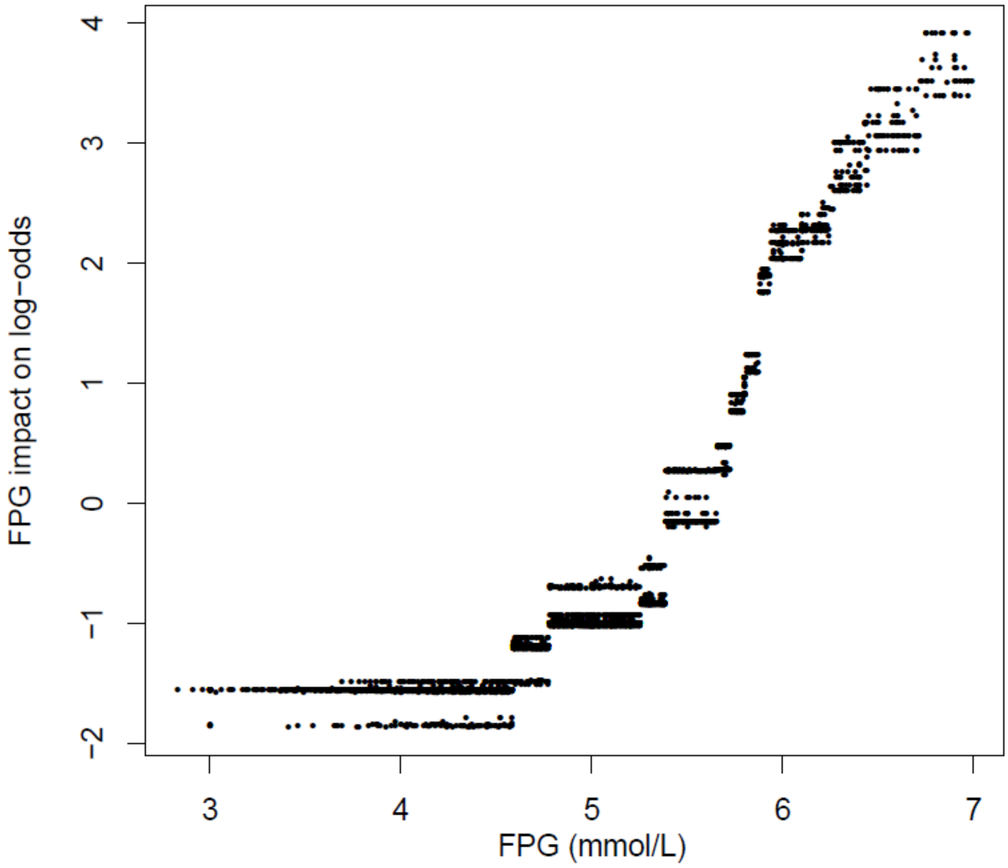

Fig S2

B

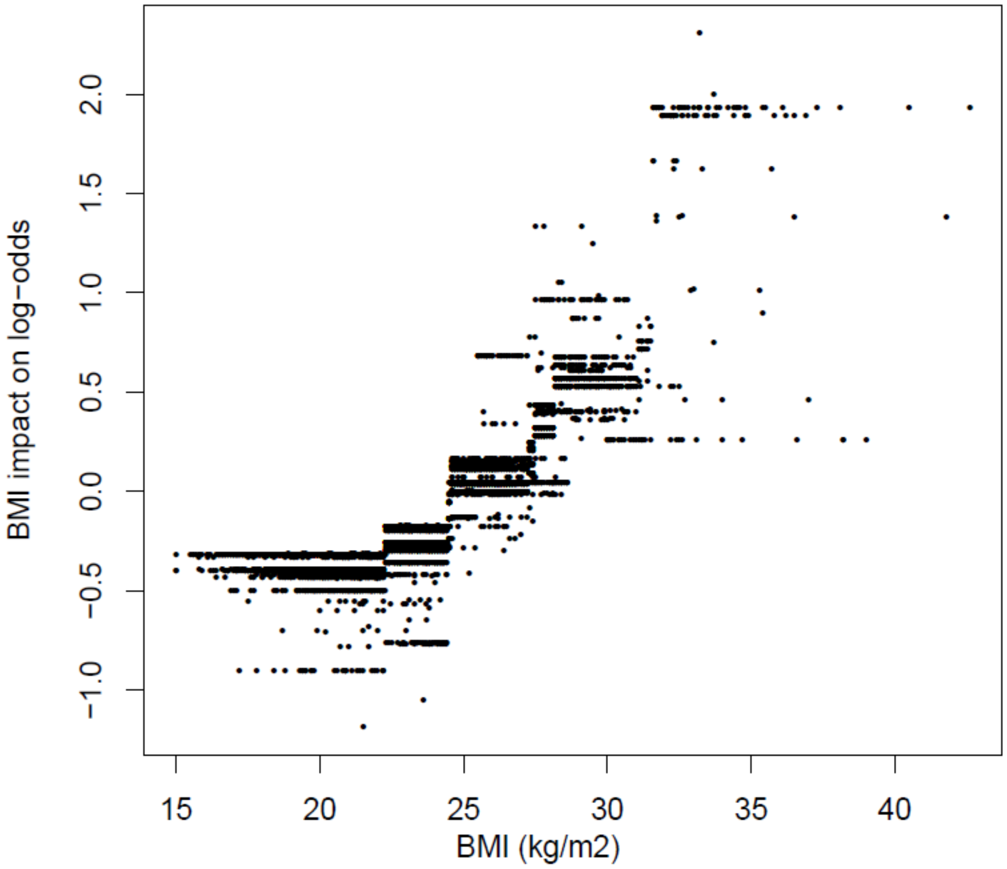
BMI impact on log−odds
−1.0
−0.5
0.0
0.5
1.0
1.5
2.0
15
20
25
30
35
40
BMI (kg/m2)

Fig S2
C

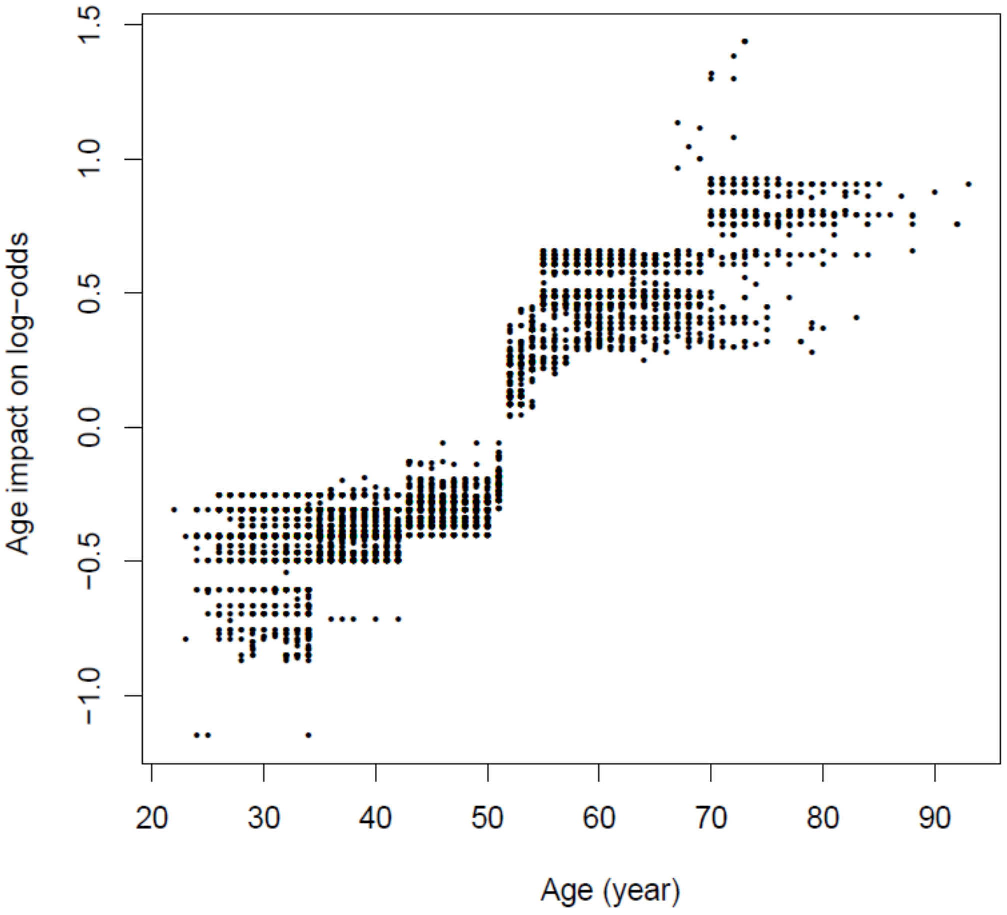

Fig S2. Three examples of dependence plots based on the SHAP approach, showing predicted relative risk versus feature value for FPG (a), BMI(b) and age (c). Each black dot represents the effect of each participant's independent variable on incident diabetes, which is related to interactions between input features.

Fig S3

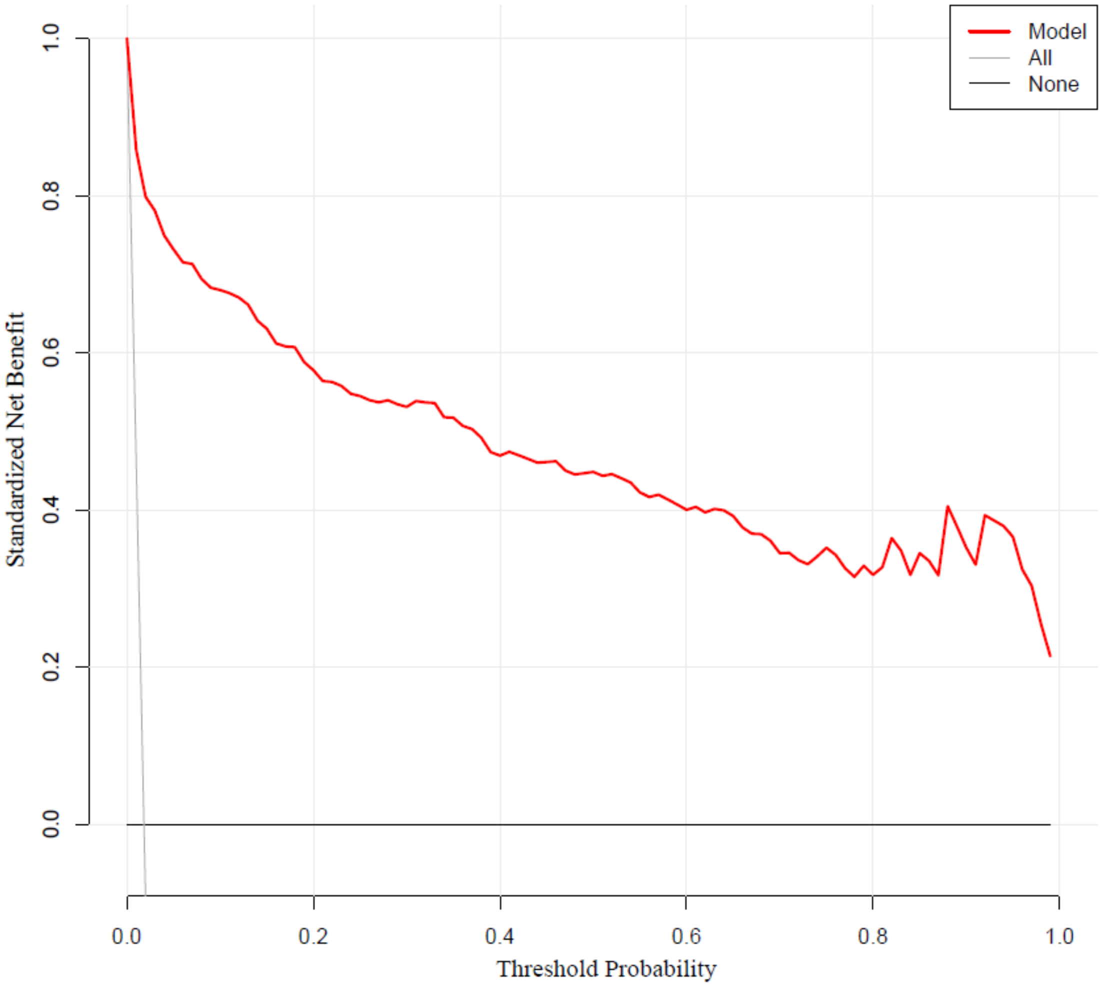

Fig S3. Decision curve for the XGBoost model to predict incident diabetes. Net benefit is shown on the y-axis. The red line represents the model; the thin gray line represents the assumption that all participants develop diabetes; the thin black line represents the assumption that none participants develop diabetes. The decision curve demonstrated that if the threshold probability of a patient is > 1%, using the model to predict incident diabetes adds more benefit than diabetes screenings (i.e., oral glucose tolerance test) for all or none of participants. For example, if the personal threshold probability of a participant is 50% (i.e., the participant would opt for diabetes screening if the probability of incident diabetes was < 50%), then the net benefit is 0.453 when using the model to make the decision of whether to perform diabetes screening, with added benefit compared to the diabetes screening for all or none participants.

Fig S4

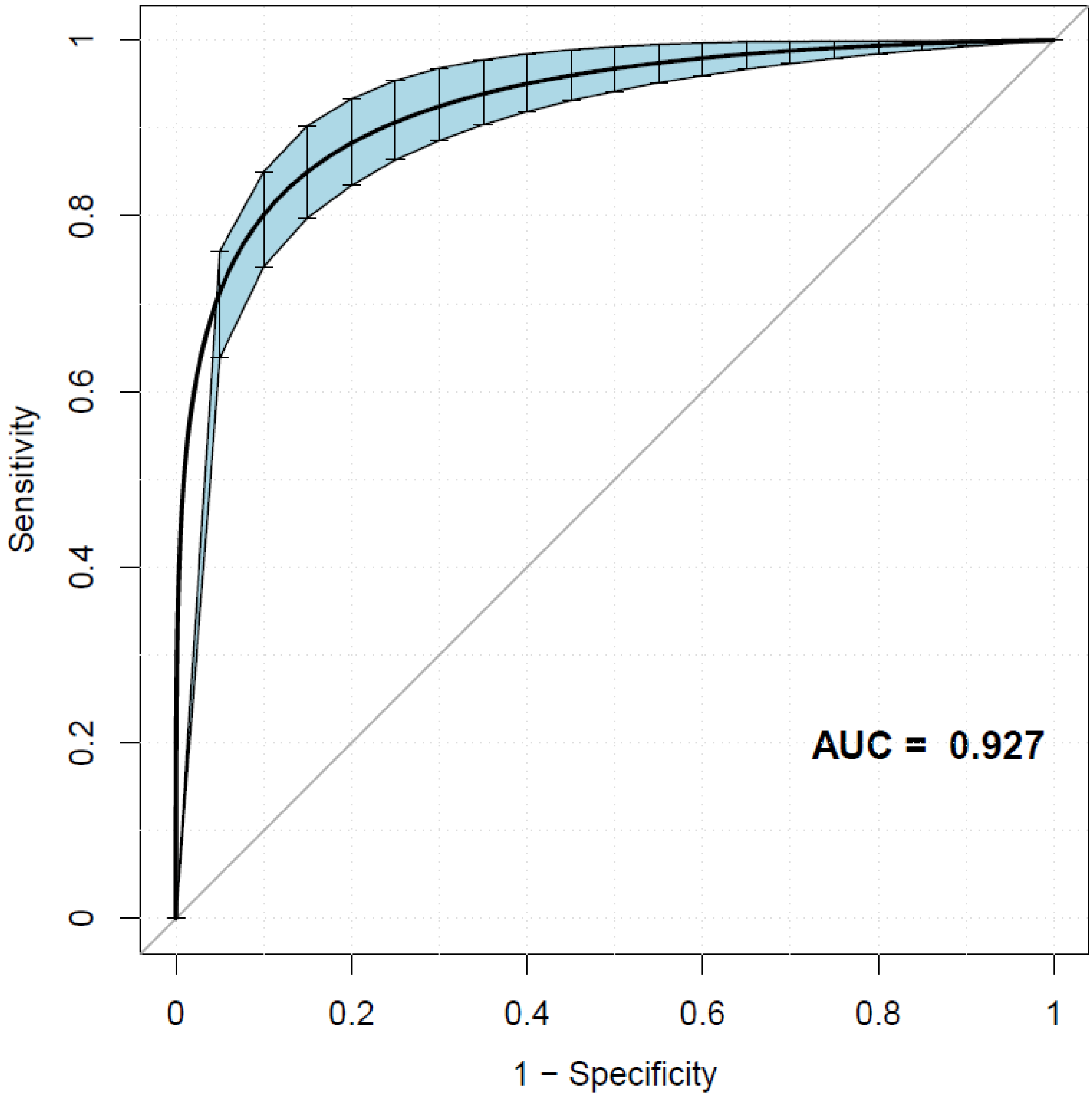

Fig S4. Using bootstrap resampling validation (times=500) to confirm the prediction performance stability of the nomogram of the stepwise model in the training cohort.
